# Some New Maximally Chaotic Discrete Maps

**DOI:** 10.3390/e28010131

**Published:** 2026-01-22

**Authors:** Hyojeong Choi, Gangsan Kim, Hong-Yeop Song, Sangung Shin, Chulho Lee, Hongjun Noh

**Affiliations:** 1 Department of Electrical and Electronic Engineering, Yonsei University, Seoul 03722, Republic of Korea; hjchoi3022@yonsei.ac.kr (H.C.); gs.kim@yonsei.ac.kr (G.K.); 2 Department of C4I R&D Center, LIG Nex1, 333 Pangyo-ro, Bundang-gu, Seongnam-si 13488, Republic of Korea; sangung.shin@lignex1.com (S.S.); chulho.lee2@lignex1.com (C.L.); hongjun.noh@lignex1.com (H.N.)

**Keywords:** chaotic map, finite precision, discrete chaos, skew tent map, discrete Lyapunov exponent, random sequences

## Abstract

In this paper, we first prove (Theorem 1) that any two inputs producing the same output in a symmetric pair of discrete skew tent maps always have the same parity, meaning that they are either both even or both odd. Building on this property, we then propose (Definition 1) a new discrete chaotic map and prove that (Theorem 2) the proposed map is a bijection for all control parameters. We further prove that (Theorem 3) the discrete Lyapunov exponent (dLE) of the proposed map is not only positive but also approaches the maximum value among all permutation maps over the integers $\left\{0,1,\ldots ,2^{m}-1\right\}$ as *m* gets larger. In other words, (Corollary 1) the proposed map asymptotically achieves the highest possible chaotic divergence among the permutation maps over the integers $\left\{0,1,\ldots ,2^{m}-1\right\}$. To provide some further evidence that the proposed map is highly chaotic, we present at the end some results from the numerical experiments. We calculate the approximation and permutation entropy of the output integer sequences. We also show the NIST SP800-22 tests results and correlation properties of some derived binary sequences.

## 1. Introduction

Chaos has attracted significant attention in various engineering fields due to its inherent properties such as sensitivity to initial conditions, aperiodic behavior, and broadband spectrum. In particular, chaotic systems have been widely utilized in cryptography [1,2,3,4], communication systems [5,6,7], and pseudo-random number generation (PRNG) [3,8,9,10,11], where their intrinsic complexity and unpredictable behavior play a crucial role in enhancing security and improving randomness.

Conventional chaotic maps are defined over the real field. When such maps are implemented on digital hardware, the inherent dynamical properties are inevitably degraded due to the limitations of finite precision [12,13,14,15,16,17,18,19,20]. In the finite-precision domain, rounding and truncation errors accumulate over iterations, resulting in the disturbances in the generated trajectories. This issue is particularly critical for chaotic systems, because even some initially close trajectories diverge after some iterations due to the sensitivity to the initial conditions, which is quantified by the Lyapunov exponent [12]. Moreover, the extent of degradation varies with the computational precision of the hardware [13,15,21], preventing the preservation of the expected chaotic behavior in the digital domain. As a result, when a chaotic map is implemented digitally, it may suffer from the finite-precision effects. This may result in not only the generated trajectories to differ from the ideal chaotic behavior but also some of their statistical properties modified [20].

To address these limitations and enable practical applications of chaos, several techniques have been proposed to enhance the robustness of chaotic maps in the digital domain [3,16,17,19,22]. For example, ref. [17,22] proposed a perturbation framework to disturb chaotic orbits and avoid extreme dynamic degradation. In ref. [3], least significant bit extension was applied to the binary shift chaotic map to generate true chaotic orbits under finite precision. Finally, ref. [16,19] analyzed the impact of digital circuits on chaotic systems and introduced a control term to improve periodic orbits.

Meanwhile, to clarify the notion of chaos in digital environments, several studies have focused on defining chaos in the discrete domain [23,24,25]. For example, ref. [23] demonstrated that even when a continuous chaotic system is quantized into a semi-digital form, it can still satisfy Devaney’s definition of chaos under certain conditions. In addition, ref. [24,25] introduced the Discrete Lyapunov Exponent (dLE) as a reformulation of the conventional Lyapunov exponent for discrete systems. These works showed that when the size of the discrete space tends to infinity, a positive dLE implies chaotic behavior, and emphasized that bijective structures can eliminate stable periodic orbits, enabling sustained chaotic dynamics.

Building on these foundations, various studies have proposed new chaotic maps in discrete spaces and analyzed their dynamical behavior. These studies primarily evaluate whether the proposed maps exhibit chaotic properties using the dLE [9,14,26,27,28,29]. For example, ref. [9] introduced a digitalized modified logistic map, while ref. [14] proposed a discrete chaotic map family based on the discrete Arnold Cat Map over integer rings. In [29], it was shown that the integer sequences derived from interpreting the states of a primitive LFSR exhibit chaotic behavior. This is further generalized to the case where the connection polynomial is irreducible [30]. In addition, various discrete chaotic systems have been proposed, including those based on the composition of permutations [27], as well as integer- and finite-field-based constructions [26,28]. Discrete chaotic maps defined in the discrete domain play a vital role in cryptography and pseudorandom number generation, and continued research is required to support their broader use in various digital applications.

The skew tent map is known to exhibit chaotic behavior over the entire range of control parameters t∈(0,1), and owing to this property, it has been widely applied in various fields [1,31,32,33,34]. However, despite this advantage, under finite-precision implementation, binary sequences generated by a single skew tent map can pass the NIST SP800-22 tests only when *t* is extremely close to 0.5, and their correlation properties are also unsatisfactory [31]. This ultimately serves as a limitation that restricts the inherent advantage of exploiting the entire range of t∈(0,1), thereby diminishing the potential applications of skew tent map-based sequences [31].

In this paper, we introduce a new discrete chaotic map which leverages an interesting structural property of the digitalized skew tent map. The proposed map satisfies the definition of discrete chaos for all control parameters and, as the size of the discrete space increases, its dLE approaches the maximal value attainable in the same domain. It can therefore be regarded as a maximally chaotic map as defined in [25,35], meaning that it exhibits the most chaotic behavior among maps defined over the same discrete space. Furthermore, numerical experiments confirm that the proposed map generates integer sequences with high complexity, and the derived binary sequences also demonstrate superior complexity and correlation properties, validating their potential use in various applications.

The remainder of this paper is organized as follows. Section 2 introduces the proposed map. Section 3 investigates its chaotic behavior through dLE analysis. Section 4 presents numerical experiments demonstrating the improved correlation and randomness properties of the proposed map compared to the skew tent map. Finally, Section 5 discusses some concluding remarks for future research.

## 2. Discrete Skew Tent Map and the Proposed Map

**Figure 1:**
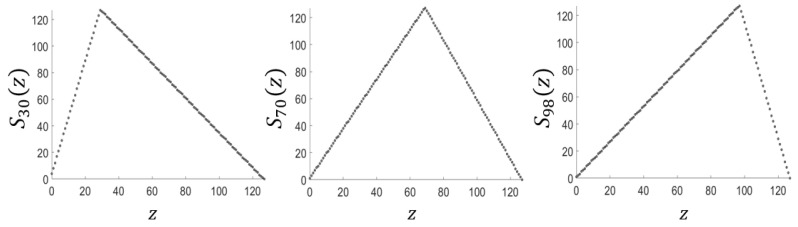
Phase portraits of $S_{a}$ with the precision m=7 and a=30, a=70 and $a=2^{7}-30=98$.

The original skew tent map [1,24,31] is defined over the interval [0,1] of real numbers as follows:

(1) $$f_{t}\left(x\right)=\left\{\begin{array}{cc} \frac{x}{t}, & 0<x\leq t,\\ \frac{1-x}{1-t}, & t<x\leq 1, \end{array}\right.$$

where 0<t<1 is a control parameter. It is known to exhibit chaotic behavior for all values of *t* [1,31]. When $f_{t}\left(x\right)$ is discretized with *m*-bit precision, it can be redefined as a discrete skew tent map, which is a bijection over the integers in the range $\left[0,2^{m}\right)$, and can be expressed as follows [1]:

(2) $$S_{a}\left(z\right)=\left\{\begin{array}{cc} \left\lceil \frac{z+1}{(\left(a/2^{m}\right)}\right\rceil -1, & 0\leq z<a,\\ \left\lfloor \frac{2^{m}-\left(z+1\right)}{1-(a/2^{m})}\right\rfloor , & a\leq z<2^{m}, \end{array}\right.$$

where $0<a<2^{m}$ is an integer control parameter. Figure 1 shows the phase portrait of the discrete skew tent map with m=7 for the various values of the control parameter *a*.

**Theorem** **1.** *Let $a\in (0,2^{m})$ be an integer. Let $S_{a}$ and $S_{2^{m}-a}$ denote the discrete skew tent maps defined in* (2) *with the control parameters a and $2^{m}-a$, respectively. For any two integers $z,z^{\prime }\in \left[0,2^{m}-1\right]$, if $S_{a}\left(z\right)=S_{2^{m}-a}\left(z^{\prime }\right)$, then* $z\equiv z^{\prime }$ (mod 2).

**Proof.**  For convenience, we represent the control parameter as $a=\delta 2^{k}$, where δ>0 is an odd integer and 0≤k<m. We rewrite $S_{\delta 2^{k}}\left(z\right)$ and $S_{2^{m}-\delta 2^{k}}\left(z^{\prime }\right)$ as follows:

$$S_{\delta 2^{k}}\left(z\right)=\left\{\begin{array}{cc} \left\lceil \frac{\left(z+1\right)2^{m-k}}{\delta }\right\rceil -1, & 0\leq z<\delta 2^{k},\\ \left\lfloor \frac{\left(2^{m}-\left(z+1\right)\right)2^{m-k}}{2^{m-k}-\delta }\right\rfloor , & \delta 2^{k}\leq z<2^{m}, \end{array}\right.$$

and

$$S_{2^{m}-\delta 2^{k}}\left(z^{\prime }\right)=\left\{\begin{array}{cc} \left\lceil \frac{\left(z^{\prime }+1\right)2^{m-k}}{2^{m-k}-\delta }\right\rceil -1, & 0\leq z^{\prime }<2^{m}-\delta 2^{k},\\ \left\lfloor \frac{\left(2^{m}-\left(z^{\prime }+1\right)\right)2^{m-k}}{\delta }\right\rfloor , & 2^{m}-\delta 2^{k}\leq z^{\prime }<2^{m}. \end{array}\right.$$

Now, we will go through all the values of *z* in the first equation of $S_{\delta 2^{k}}$ in the range $0\leq z<\delta 2^{k}$ or $1\leq z+1<\delta 2^{k}+1$. Due to the ceiling function, we distinguish these values of z+1 into two cases. Case 1 is where z+1 is a multiple of δ and Case 2 is where z+1 is not a multiple of δ.

- **Case 1.** We consider the values of z+1=iδ in the first equation of $S_{\delta 2^{k}}$ for some *i*. Then the values of $S_{\delta 2^{k}}$ will match with those of the first equation of $S_{2^{m}-\delta 2^{k}}$ corresponding to the input $z^{\prime }+1=i\left(2^{m-k}-\delta \right)$. Therefore, both z+1=iδ and
  $$z^{\prime }+1=i\left(2^{m-k}-\delta \right)=i2^{m-k}-\left(z+1\right)$$
  must have the same parity. We note that then the value $i2^{m-k}-1$ from $S_{\delta 2^{k}}$ will not have any match with the second equation of the map $S_{2^{m}-\delta 2^{k}}$ above since $S_{2^{m}-\delta 2^{k}}$ is a bijection.
- **Case 2.** We consider the values of z+1 that is not a multiple of δ in the first equation of $S_{\delta 2^{k}}$, and observe in this case that
  $$\left\lceil \frac{\left(z+1\right)2^{m-k}}{\delta }\right\rceil -1=\left\lfloor \frac{\left(z+1\right)2^{m-k}}{\delta }\right\rfloor .$$
  This time, the value above can be matched with some values from the second equation of the map $S_{2^{m}-\delta 2^{k}}$ above. Now, assume that
  $$\left\lfloor \frac{\left(z+1\right)2^{m-k}}{\delta }\right\rfloor =\left\lfloor \frac{\left(2^{m}-\left(z^{\prime }+1\right)\right)2^{m-k}}{\delta }\right\rfloor ,$$
  and z+1≢0(modδ). Then, we have $z+1=2^{m}-\left(z^{\prime }+1\right)$ or $z^{\prime }=2^{m}-\left(z+2\right)$, and both *z* and $z^{\prime }$ have the same parity. Note again that $S_{2^{m}-\delta 2^{k}}$ is a bijection. Therefore, since any of the values from the first equation of $S_{\delta 2^{k}}$ for z+1≢(modδ) has been matched with the second equation of $S_{2^{m}-\delta 2^{k}}$, it cannot be matched with the first equation of $S_{2^{m}-\delta 2^{k}}$.

Remaining cases are the values of the input to the second equation of $S_{\delta 2^{k}}$. The cases when the output match with either the first or the second equation of the map $S_{2^{m}-\delta 2^{k}}$ can be done similarly. □

Recall that *z* and $z^{\prime }$ always have the same parity if $S_{a}\left(z\right)=S_{2^{m}-a}\left(z^{\prime }\right)$. Therefore, by picking up any one of $S_{a}$ and $S_{2^{m}-a}$ for all even inputs and picking up the other for all odd inputs, we may construct a new bijection over the same set of integers in the range $\left[0,2^{m}\right)$.

**Definition** **1.** *For $z=0,1,\ldots ,2^{m}-1$, we define a map $C_{a}$ as*

(3) $$C_{a}\left(z\right)=\left\{\begin{array}{cc} S_{a}\left(z\right), & \mathrm{if}\;z\;\mathrm{is}\;\mathrm{even},\\ S_{2^{m}-a}\left(z\right), & \mathrm{if}\;z\;\mathrm{is}\;\mathrm{odd}, \end{array}\right.$$

*where $0<a<2^{m}$ is a given control parameter.*

**Figure 2:**
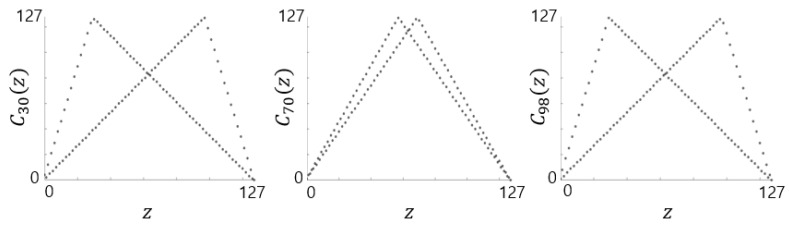
Phase portraits of $C_{a}$ with the precision m=7 and a=30, a=70 and $a=2^{7}-30=98$.

Figure 2 shows the phase portraits of the proposed map with m=7 for three different control parameters. Interestingly, the phase portraits for a=30 and a=98 (=128 − 30) appear visually similar, yet they produce completely different output sequences. This difference in output behavior is further supported by their low cross-correlation values, as discussed in Section 4.4.

**Figure 3:**
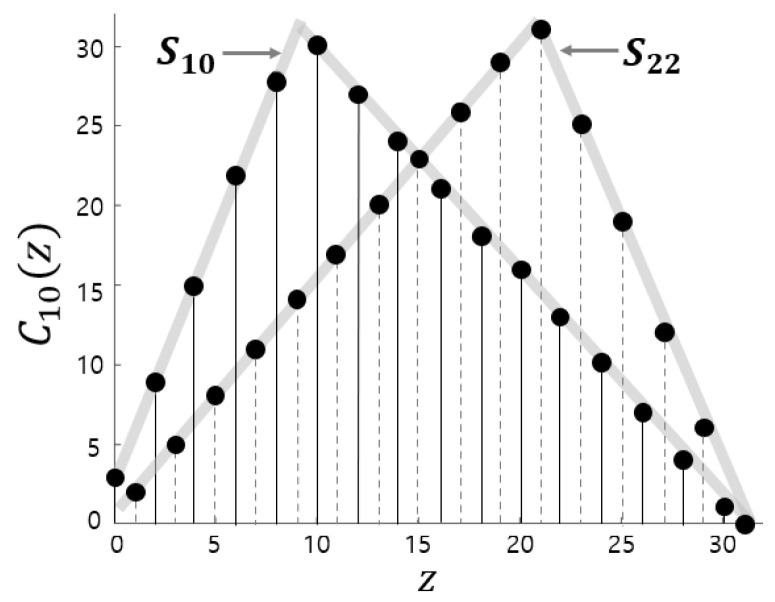
Proposed map $C_{10}$ with m=5 from $S_{10}$ and $S_{22}$.

Figure 3 illustrates how the proposed map is constructed according to Definition 1. In this example, $C_{10}$ for m=5 is formed by sampling $S_{10}\left(z\right)$ at even indices and $S_{22}\left(z\right)$ at odd indices, visually demonstrating the piecewise composition of the map. We note that when $a=2^{m-1}$, the pair $S_{a}$ and $S_{2^{m}-a}$ coincide, and the proposed map becomes the same as the discrete skew tent map with $a=2^{m-1}$, which is in fact the discrete tent map.

**Theorem** **2.** *For any control parameter $0<a<2^{m}$, the proposed map $C_{a}$ defined in* (3) *is a bijection over the integers in the range $\left[0,2^{m}-1\right]$.*

**Proof.**  The $C_{a}$ in (3) is constructed by combining two symmetric skew tent maps $S_{a}$ and $S_{2^{m}-a}$ by taking $S_{a}\left(i\right)$ for only even *i* and taking $S_{2^{m}-a}\left(i\right)$ for only edd *i*. Therefore, the input domain remains the same as $\left[0,2^{m}-1\right]$. Recall that both $S_{a}$ and $S_{2^{m}-a}$ are bijections over the same range $\left[0,2^{m}-1\right]$. By Theorem 1, when restricted to even and odd inputs, the output ranges of $S_{a}$ and $S_{2^{m}-a}$ are disjoint and now their union becomes the range $\left[0,2^{m}-1\right]$. □

## 3. Chaotic Behavior of the Proposed Map

We now analyze the chaotic behavior of the proposed map. In general, a permutation *F* is said to be *discretely chaotic* if its dLE satisfies [24,25]

(4) $$\lim_{M\to \infty }\lambda_{F}>0,$$

where *M* is the size of the discrete domain of *F*.

**Theorem** **3.** *Let m be a positive integer and consider the proposed map $C_{a}$ with control parameter $a\in (0,2^{m})$. Then, the discrete Lyapunov exponent $\lambda_{C_{a}}$ asymptotically approaches mln(2) as m increases, for all a, except for $a=2^{m-1}$.*

The dLE of $C_{a}$ is known to be ln(2) when $a=2^{m-1}$ [24].

**Proof.**  To clarify the notion of neighborhood in a discrete ordered set, consider the domain $M:=\{0,1,2,\ldots ,2^{m}-1\}$ of the map $C_{a}$. Here, the neighbors of i∈M are i−1 and i+1 for $i=2,3,\ldots ,2^{m}-2$. It is to be noted that 0∈M has only one neighbor 1, and $2^{m}-1\in M$ also has only one neighbor $2^{m}-2$. Then, the dLE $\lambda_{C_{a}}$ of a permutation $C_{a}$ on the set M is defined as follows [24,25]:

(5) $$\lambda_{C_{a}}=\frac{1}{2^{m}}\sum_{i=0}^{2^{m}-1}\ln\left|C_{a}\left(z_{i+1}\right)-C_{a}\left(z_{i}\right)\right|,$$

where $z_{i}=i$ for i∈M and $z_{2^{m}}=2^{m}-2$.

According to the definition of the dLE in (5), it is computed by averaging the logarithmic differences between the outputs of neighboring input values over the domain. To analyze how these differences behave for the proposed map $C_{a}\left(z\right)$ as *m* increases, we introduce a normalized real parameter t∈(0,1), which is independent of *m*, and express the control parameter *a* in terms of *t* as

$$a=\lfloor t\;2^{m}\rfloor .$$

**Figure 4:**
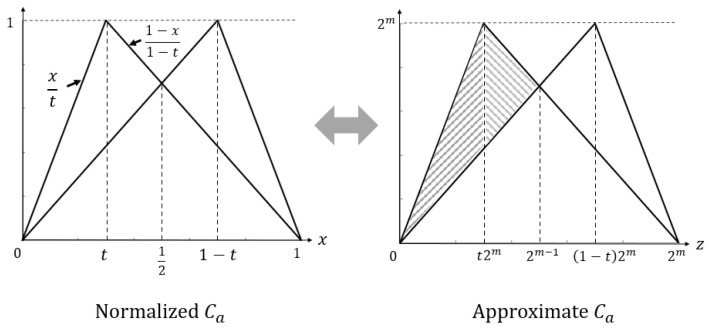
Normalized $C_{a}$ and approximate $C_{a}$.

Then, the normalized proposed map is a combination of a pair of symmetric skew tent maps in (1), which is shown in Figure 4. Note that t=0.5 corresponds to the control parameter $a=2^{m-1}$ which is not considered here. Accordingly, we focus on the parameter range t∈(0,1)∖{0.5} in the proof.

Then the value $2^{m}\lambda_{C_{a}}$ in (5) becomes approximately the area under the natural log of the absolute difference between two lines in the figure. Since the left and right are symmetric, we only have to calculate the left part (shaded part) and double the result. It consists of two parts: 0≤x≤t and t≤x<1/2, where *x* is the the normalized real variable corresponding to the discrete variable *z* by the relation

$$z=\lfloor x\;2^{m}\rfloor .$$

Then, for 0≤x≤t, the difference becomes

$$\frac{x}{t}-\frac{x}{1-t}=x\frac{1-2t}{t(1-t)}.$$

For t≤x<1/2, the difference becomes

$$\frac{1-x}{1-t}-\frac{x}{1-t}=\frac{1-2x}{1-t}.$$

Therefore, the value $\lambda_{C_{a}}$ in (5) can now be computed using the approximate $C_{a}$ in Figure 4 as follows:

$$\begin{array}{cc} \lambda_{C_{a}} & \approx \frac{2}{2^{m}}\left(\int_{0}^{t2^{m}}\ln\left(z\frac{1-2t}{t(1-t)}\right)dz\right.+\left.\int_{t2^{m}}^{2^{m-1}}\ln\left(\frac{1-2z}{1-t}\right)dz\right)\\ & \approx m\ln(2), \end{array}$$

as *m* gets larger and larger. □

**Remark** **1.** *The largest dLE $\lambda_{F_{\max}}$ has been derived for all permutations over the discrete phase space {0,1,…,M−1}, where M is an even integer [25,35]. When $M=2^{m}$, the largest dLE $\lambda_{F_{\max}}$ is given by*

(6) $$\;\;\lambda_{F_{\max}}\left(m\right)\;=\;\frac{2^{m-1}+\;1}{2^{m}}\ln2^{m-1}\;+\frac{2^{m-1}-\;1}{2^{m}}\ln\left(2^{m-1}+\;1\right).$$

*The permutations that achieve this largest $\lambda_{F_{\max}}$ are referred to as maximal discrete chaotic maps, as they possess the largest possible dLE among all permutations on phase spaces of the same size [25,35]. It is easy to see that $\lambda_{F_{\max}}\left(m\right)\to m\ln\left(2\right)$ as m gets larger and larger. We extend this notion and define the asymptotic version of this maximal chaos.*

**Definition** **2.** *A discrete chaos map F is called asymptotically maximally discrete chaotic if its dLE $\lambda_{F}>0$ satisfies*

$$\lim_{m\to \infty }\frac{\lambda_{F}}{\lambda_{F_{\max}}}=1,$$

*where $\lambda_{F_{\max}}$ is given in* (6).

**Corollary** **1.** *The proposed map $C_{a}$ for any control parameter $a\in (0,2^{m})$∖$\left\{2^{m-1}\right\}$ is asymptotically maximally discrete chaotic.*

**Figure 5:**
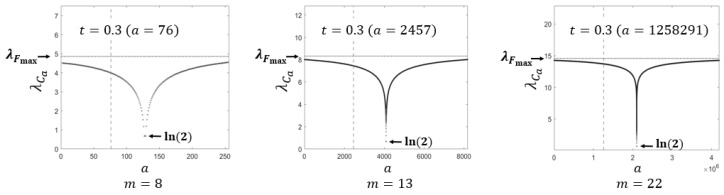
The dLE of $C_{a}$ with precision m=8, m=13 and m=22.

Figure 5 compares the dLE $\lambda_{C_{a}}$ of the proposed map with the maximal dLE $\lambda_{F_{\max}}$ among all permutations over the set $\left\{1,2,\ldots ,2^{m}-1\right\}$, computed from (6), for m=8, m=13 and m=22. The horizontal dashed line marks the maximal dLE $\lambda_{F_{\max}}$ for each *m*, and the vertical dashed line indicates the location of the control parameter $a=\lfloor 0.3\cdot 2^{m}\rfloor$ corresponding to t=0.3. Notably, the local minimum of $\lambda_{C_{a}}$ always occurs at $a=2^{m-1}$, and this value equals ln(2), consistent with the dLE of the symmetric skew tent map $S_{2^{m-1}}$ or the proposed map $C_{2^{m-1}}$ as previously mentioned. This figure illustrates how the ratio $\lambda_{C_{a}}/\lambda_{F_{\max}}$ gradually approaches 1 as *m* increases, as indicated by Corollary 1.

## 4. Numerical Simulations

### 4.1. Bifurcation Diagrams

The bifurcation diagram is a graphical tool used to visualize the dynamic behavior of a chaotic system. It shows how the output states of the chaotic map are distributed as its control parameter varies within a given interval [7].

**Figure 6:**
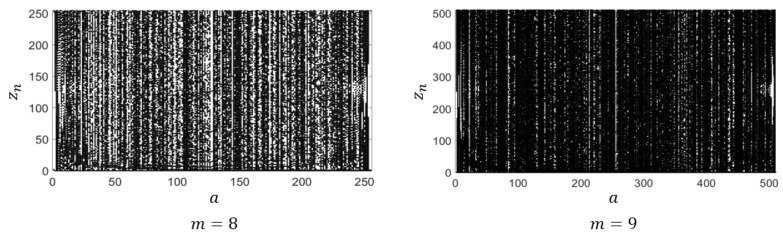
Bifurcation diagrams of $C_{a}$ for m=8 and m=9 with the initial value $z_{0}=0$.

Figure 6 presents the bifurcation diagrams of the proposed map for m=8 and m=9, obtained by iterating the map from the initial state $z_{0}=0$. For each control parameter *a*, the integer states $z_{n}$ are plotted to illustrate how the distribution evolves as *a* varies. As shown in both cases of Figure 6, the state points are densely and almost uniformly dispersed over the entire phase space, forming a distribution that nearly covers the full interval $\left[0,2^{m}-1\right]$. Although a few short-period cycles appear for certain control parameters, these are dependent on the initial condition; for most cases, the trajectories generated from $z_{0}=0$ still occupy the entire state space densely. Therefore, the bifurcation diagrams confirm that the proposed map maintains nearly ergodic and well-dispersed behavior over a broad parameter range.

### 4.2. Complexity of Integer Sequences

To evaluate the complexity of integer sequences generated by the proposed map and the discrete skew tent map, we employ two widely used entropy-based measures: approximate entropy (ApEn) [36] and permutation entropy (PE) [37].

ApEn quantifies the regularity and unpredictability of a time series by estimating the logarithmic likelihood that similar patterns of length *L* remain similar within a tolerance *r* when extended to length L+1 [36]. It is then computed as the logarithmic difference between the average probabilities of similarity for pattern lengths *L* and L+1. In this evaluation, we adopt commonly used parameters L=2 and r=0.2σ, where σ denotes the standard deviation of the sequence and *r* serves as the threshold for determining whether two subsequences are considered similar [36]. A higher ApEn value indicates greater complexity and lower predictability, which are desirable characteristics of chaotic sequences.

PE quantifies the complexity of a time series by evaluating the distribution of ordinal patterns formed by subsequences of length *L* separated by an embedding delay *D* [37]. Each subsequence is ranked in ascending order, and the relative frequencies of all possible order permutations are computed to obtain a normalized Shannon entropy value. In this evaluation, we adopt the commonly used parameters L=6 and D=2, as suggested in [22,37]. Larger PE values imply more uniformly distributed ordinal patterns, reflecting higher dynamical complexity and stronger chaotic behavior.

**Figure 7:**
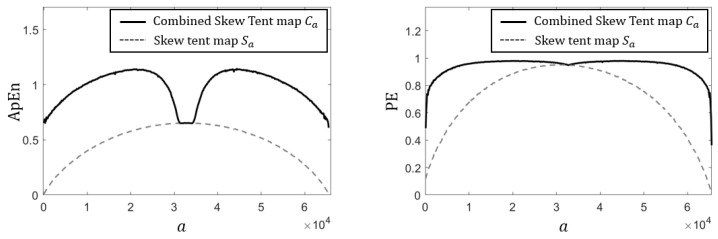
Comparison of ApEn and PE of integer sequences.

We consider the case of m=16, for which the control parameter *a* ranges over (0, 65,536), and compute ApEn and PE for the output sequences. The results, presented in Figure 7, confirm that the proposed map consistently produces significantly more complex and less predictable integer sequences than the discrete skew tent map.

### 4.3. Randomness of Binary Sequences

We use the NIST SP800-22 statistical test suite [38], which comprises 15 sub-tests conducted at a significance level of α=0.01. A test is considered passed if its pass rate is at least 0.96 [38].

The simplest method for generating binary sequences is the threshold-based conversion, which directly reflects the statistical bias of the map’s time series. It is well known that discrete skew tent maps fail to pass the NIST tests under this method [31]. To examine whether the proposed map exhibits such bias, we first generated binary sequences using the threshold-based method and evaluated them with the NIST test suite.

In this threshold-based approach, binary sequences are generated by iterating $C_{a}$ and mapping each state $x_{n}\in \left\{0,1,\ldots ,2^{m}-1\right\}$ to 0 if $x_{n}<2^{m-1}$ and to 1 otherwise. We set m=32 and extract 100 subsequences of length $10^{6}$ from a single sequence of length $10^{8}$. Table 1 reports the pass rates of each sub-test for binary sequences generated by $C_{a}$, where $a=\lfloor t2^{m}\rfloor$ for various values of *t*. For sub-tests with multiple components (marked with an *), the minimum proportion is reported. As shown in Table 1, the proposed map passes all NIST tests for every tested value of *a*, suggesting that its output sequences are statistically well balanced.

To further validate the randomness of the proposed map, we generated binary sequences using an XOR-based conversion under the same experimental conditions as the threshold-based case. In this approach, a new binary sequence was obtained by performing a three-bit XOR operation across different bit positions of each *m*-bit integer output, specifically the 1st, (m−1)-th, and *m*-th bits. Table 2 presents the NIST test results for the XOR-based sequences generated from the proposed map with m=32. As shown, these sequences also pass all NIST tests for every tested value of *a*, indicating that the generated binary sequences exhibit strong statistical randomness.

### 4.4. Correlation Analysis of Binary Sequences

Auto-correlation and cross-correlation are key metrics for evaluating the dependency structure in (binary) sequences from chaotic maps [19,31]. In this subsection, we examine these properties for the proposed map by generating binary sequences using the same binary mapping rules described in the previous subsection.

**Figure 8:**
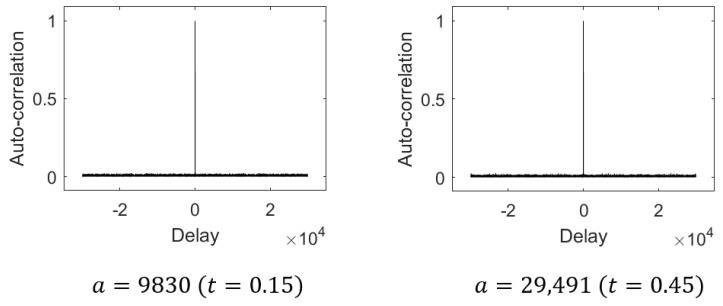
Auto-correlation of some binary sequences from the proposed map.

Figure 8 shows the auto-correlation results for binary sequences generated from the proposed map using the threshold-based conversion. Each sequence has a length of 30,000 with m=16, and two representative control parameters were tested: a=9830 (left) and *a* = 29,491 (right). In both cases, the maximum side-lobe values remain approximately 0.0225. Under the same parameters, the XOR-based sequences exhibited a comparable maximum side-lobe value of approximately 0.0237.

**Figure 9:**
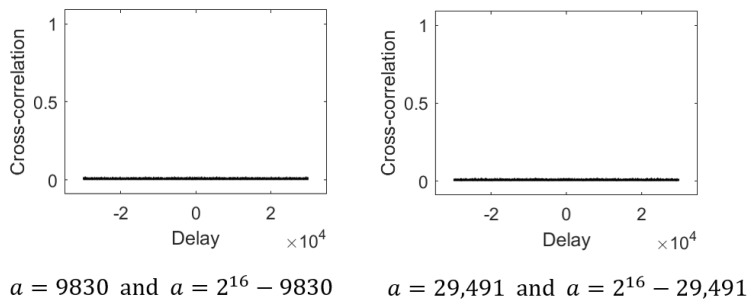
Cross-correlation of some binary sequences from two proposed maps.

As mentioned earlier, two proposed maps $C_{a}$ and $C_{2^{m}-a}$ have visually similar phase portraits (Figure 2 for m=7 and a=30), but they are in fact distinct in detail. Figure 9 shows the cross-correlation between sequences generated from the two proposed maps: a=9830 and $2^{16}-9830$ (left), and *a* = 29,491 and $2^{16}$− 29,491 (right), both with m=16. The maximum cross-correlation values are 0.022 and 0.023, respectively, indicating sufficiently low inter-sequence correlations.

The current result is only some supporting evidence to demonstrate that binary sequences generated from the proposed map through some simple conversion rules can still exhibit reasonably good correlation properties in practice. The proof or the optimization of the correlation performance is beyond the scope of the present work. We note further that the correlation characteristics may be improved by employing more sophisticated binary mapping rules.

## 5. Concluding Remarks

In this paper, we proposed the new discrete chaotic map derived from a pair of symmetric discrete skew tent maps. We proved that it is bijective and asymptotically maximally discrete chaotic. We also conducted computational analyses on both the dynamical behavior of the map and the complexity of its derived sequences. The results demonstrate that the proposed map exhibits desirable characteristics such as uniformity, unpredictability, strong randomness, and low correlation. These properties make the proposed map particularly suitable for PRNG design, where statistical balance and unpredictability are essential, and also indicate its potential applicability to cryptography, secure communications, digital watermarking, and other information-security-related systems.

Periodicity of output sequences is another fundamental aspect of discrete dynamical systems. While some empirical observations have been made, a comprehensive understanding of how the period length behaves within the parameter space of the proposed map is still lacking and could be further explored in future work. As illustrated in the bifurcation diagrams, most control parameters yield long-period trajectories, whereas short cycles occasionally appear for specific control parameters and initial conditions. A more systematic investigation is therefore required to clarify how the period length depends on the control parameters, initial conditions, and the structure of the discrete state space.

Furthermore, the proposed map is based on a symmetric two-piecewise structure, and its extension to multi-piecewise forms could also be considered in future work. Although the proposed map already achieves asymptotically maximally discrete chaos, increasing the number of segments may not necessarily yield stronger chaotic behavior. Nevertheless, there remains potential to design new discrete chaotic maps within this framework by extending the discrete skew tent map to multi-piecewise forms. A rigorous theoretical investigation is therefore required to determine whether such extensions can also preserve chaotic behavior.

## Figures and Tables

**Table 1:** NIST test results for binary sequences of length $10^{8}$ from the proposed map using the threshold-based method.

Statistical Test	Proposed Map					
	t=0.15	t=0.30	t=0.45	t=0.60	t=0.75	t=0.90
Frequency	0.99	0.97	0.99	1.00	1.00	1.00
Block Freq.	0.98	1.00	1.00	0.99	1.00	1.00
Cumulative *	0.98	0.96	0.99	1.00	1.00	1.00
Runs	0.99	0.97	1.00	0.99	0.98	1.00
Longest Run	0.99	1.00	0.98	1.00	0.99	1.00
Rank	1.00	1.00	0.98	0.99	0.99	0.99
FFT	0.99	0.99	1.00	0.99	1.00	0.98
Nonoverlap. *	0.96	0.96	0.96	0.97	0.96	0.96
Overlap.	1.00	0.99	0.99	0.98	0.99	1.00
Universal	0.99	1.00	1.00	0.97	0.99	0.97
ApEn	0.98	0.98	0.99	0.96	0.99	0.98
Rand. Exc. *	1.00	0.98	0.96	0.96	0.96	0.96
Ran. Ex. Var. *	0.96	0.98	0.96	0.96	0.96	0.96
Serial *	0.97	0.98	0.99	0.98	0.97	0.99
Linear Comp.	0.97	0.99	0.98	0.99	0.99	0.99

* the minimum values of multiple tests.

**Table 2:** NIST test results for binary sequences of length $10^{8}$ from the proposed using the XOR-based method.

Statistical Test	Proposed Map					
	t=0.15	t=0.30	t=0.45	t=0.60	t=0.75	t=0.90
Frequency	1.00	0.99	0.99	0.99	0.97	0.99
Block Freq.	1.00	1.00	0.99	1.00	1.00	1.00
Cumulative *	1.00	0.99	1.00	0.99	0.98	1.00
Runs	0.98	1.00	1.00	1.00	0.98	0.98
Longest Run	1.00	1.00	0.99	1.00	1.00	1.00
Rank	0.99	1.00	1.00	0.96	1.00	1.00
FFT	1.00	0.99	1.00	1.00	1.00	0.99
Nonoverlap. *	0.97	0.97	0.97	0.96	0.98	0.97
Overlap.	1.00	0.98	0.98	0.98	1.00	1.00
Universal	1.00	0.97	0.99	0.99	0.98	0.98
ApEn	0.99	1.00	0.98	0.98	0.99	0.98
Rand. Exc. *	0.98	0.98	1.00	0.98	0.98	0.98
Ran. Ex. Var. *	0.98	0.98	0.97	0.98	0.98	0.98
Serial *	0.97	1.00	0.98	0.99	0.99	1.00
Linear Comp.	0.99	0.99	0.99	0.98	1.00	0.98

* the minimum values of multiple tests.

## Data Availability

No new data were created or analyzed in this study, data sharing is not applicable.
